# 2,3,6,7-Tetra­kis(bromo­meth­yl)naphthalene

**DOI:** 10.1107/S1600536810024311

**Published:** 2010-06-26

**Authors:** Maciej Skibiński, Vladimir A. Azov, Peter G. Jones

**Affiliations:** a Department of Chemistry, University of Bremen, Leobener Strasse NW 2C, 28359 Bremen, Germany; b Institute of Inorganic and Analytical Chemistry, Technical University of Braunschweig, Postfach 3329, 38023 Braunschweig, Germany

## Abstract

The title compound, C_14_H_12_Br_4_, crystallizes with imposed inversion symmetry. In the crystal, the mol­ecules pack in layers parallel to (10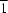). The layers involve two Br⋯Br and one H⋯Br contact. Between the layers, one contact each of types Br⋯Br, H⋯Br and Br⋯π is observed.

## Related literature

For the use of 2,3,6,7-tetra­kis­(bromo­meth­yl)naphthalene in the preparation of cyclo­phanes, see: Otsubo *et al.* (1983 ▶, 1989 ▶); Yano *et al.* (1999 ▶); Skibiński *et al.* (2009 ▶). For its applications in the synthesis of hydrogen-bonded mol­ecular capsules, see: Valdes *et al.* (1995 ▶); Rivera *et al.* (2001 ▶). For reviews on halogen–halogen contacts and ‘weak’ hydrogen bonding, see: Desiraju & Steiner (1999 ▶); Metrangolo & Resnati (2001 ▶); Metrangolo *et al.* (2008 ▶); Rissanen (2008 ▶). For the X-ray structures of the full series of ten isomeric bis­(bromo­meth­yl)naphthalenes, see: Jones & Kuś (2010 ▶). For the X-ray structures of two isomeric tetra­kis­(bromo­meth­yl)benz­ene derivatives, see: Jones & Kuś (2007 ▶).
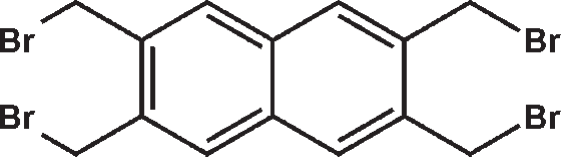


## Experimental

### 

#### Crystal data

C_14_H_12_Br_4_
*M* *_r_* = 499.88
Triclinic, 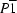

*a* = 6.6144 (2) Å
*b* = 7.1770 (2) Å
*c* = 8.7761 (3) Å
α = 84.744 (3)°
β = 78.251 (3)°
γ = 64.555 (3)°
*V* = 368.32 (2) Å^3^
*Z* = 1
Mo *K*α radiation
μ = 10.91 mm^−1^
*T* = 100 K
0.20 × 0.06 × 0.04 mm

#### Data collection

Oxford Diffraction Xcalibur Eos diffractometer
Absorption correction: multi-scan (*CrysAlis PRO*; Oxford Diffraction, 2009 ▶) *T*_min_ = 0.356, *T*_max_ = 1.000
17701 measured reflections
2122 independent reflections
1716 reflections with *I* > 2σ(*I*)
*R*_int_ = 0.031

#### Refinement

*R*[*F* ^2^ > 2σ(*F* ^2^)] = 0.016
*wR*(*F* ^2^) = 0.034
*S* = 0.92
2122 reflections
82 parameters
H-atom parameters constrained
Δρ_max_ = 0.50 e Å^−3^
Δρ_min_ = −0.49 e Å^−3^

### 

Data collection: *CrysAlis PRO* (Oxford Diffraction, 2009 ▶); cell refinement: *CrysAlis PRO*; data reduction: *CrysAlis PRO*; program(s) used to solve structure: *SHELXS97* (Sheldrick, 2008 ▶); program(s) used to refine structure: *SHELXL97* (Sheldrick, 2008 ▶); molecular graphics: *XP* (Siemens, 1994 ▶); software used to prepare material for publication: *SHELXL97*.

## Supplementary Material

Crystal structure: contains datablocks I, global. DOI: 10.1107/S1600536810024311/fk2020sup1.cif

Structure factors: contains datablocks I. DOI: 10.1107/S1600536810024311/fk2020Isup2.hkl

Additional supplementary materials:  crystallographic information; 3D view; checkCIF report

## Figures and Tables

**Table 1:** Hydrogen-bond geometry (Å, °)

*D*—H⋯*A*	*D*—H	H⋯*A*	*D*⋯*A*	*D*—H⋯*A*
C6—H6*A*⋯Br2^i^	0.99	2.96	3.7967 (17)	143
C6—H6*A*⋯Br2^ii^	0.99	2.98	3.7359 (16)	134
C5—H5⋯Br1^iii^	0.95	3.11	3.9399 (16)	147

Symmetry codes: (i) 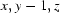; (ii) 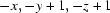; (iii) 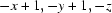.

**Table 2:** Bromine–bromine and related contacts and angles (Å, °) *Cg* is the centroid of the C1–C5,C1(−*x*, 1 − *y*, −*z*) ring.

System C—Br⋯Br—C or C—Br⋯*Cg*	Br⋯Br or Br⋯*Cg*	C—Br⋯Br (or C—Br⋯*Cg*), Br⋯Br—C	Operator
C6—Br1⋯Br2—C7	3.8972 (3)	76.45 (5), 134.79 (5)	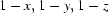
C7—Br2⋯Br2—C7	3.8873 (4)	134.93 (5) × 2	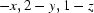
C7—Br2⋯Br2—C7	3.8913 (4)	76.72 (5) × 2	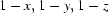
C6—Br1⋯*Cg*	3.89	158	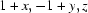

**Fig. 1.**
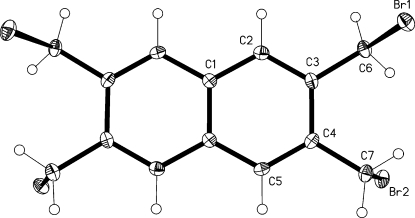
Structure of the title compound in the crystal. Ellipsoids represent 50% probability levels. Only the asymmetric unit is numbered.

**Fig. 2.**
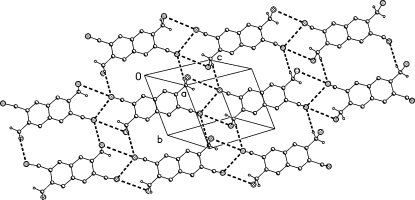
Molecular packing of the title compound as a layer parallel to (101). Br···Br and H···Br contacts are shown as thick dashed bonds.

**Fig. 3.**
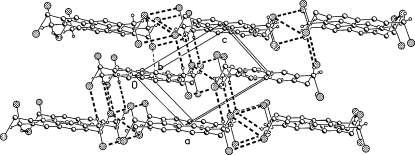
Linking between the layers of the title compound. Br···Br and H···Br contacts are shown as thick dashed bonds. One representative Br···Cg contact is shown as a thin dashed bond (top left).

## supplementary crystallographic information

### Comment

The title compound is a symmetric rigid molecule with four bromomethyl groups. The bromine atoms can be easily substituted by other nucleophiles, which offers the opportunity of employing the compound as a building block for construction of various functional architectures. The title compound was prepared to serve as a spacer between two tetrathiafulvalene (TTF) groups in TTF-containing molecular tweezers (Skibiński *et al.*, 2009). It was first employed as an intermediate in the preparation of triple-layered [2.2]naphthalenophane (Otsubo *et al.*, 1983), and several other triple-layered cyclophanes (Otsubo *et al.*, 1983; Yano *et al.*, 1999). It was also used as an intermediate in the synthesis of H-bonded molecular capsules (Valdes *et al.*, 1995; Rivera *et al.*, 2001).

The molecule of the title compound is shown in Fig. 1. It displays crystallographic inversion symmetry (operator #1 - *x*, 1 - *y*, 1 - *z*); for this reason, the crystallographic numbering does not correspond to the IUPAC numbering scheme. Bond lengths and angles may be considered normal; the bromine atoms are directed to opposite sides of the ring system, with C(2)—C(3)—C(6)—Br(1) 92.22 (16), C(3)—C(4)—C(7)—Br2 - 79.86 (17)°, which leads to a +/-/-/+ pattern of Br atoms about the ring plane for the IUPAC-numbered C2,3,6,7; this contrasts with the -/-/+/+ pattern in 1,2,4,5-tetrakis(bromomethyl)benzene (Jones & Kuś, 2007), which is also inversion-symmetric.

Details of the packing interactions are given in the Tables. The molecules pack in layers parallel to (101). Within the layers, the contacts Br1···Br2, the longer Br2···Br2 and the shorter H6A···Br2 are observed. These combine to form columns of interactions parallel to the *b* axis; chains of molecules parallel to [101] (horizontal in Fig. 2) are also formed. The contacts Br2···Br2 (the shorter), H6A···Br2 (the longer) and Br1···*Cg* (*Cg* = centre of gravity of the ring C1–5 and C1#1) link the layers (Fig. 3). H5···Br1 3.11 Å between the layers is a borderline interaction. The Br···*Cg* interaction could alternatively be interpreted as Br···C5, which at 3.450 (2) Å is by far the shortest of the six Br···C contacts; it is often unclear which is the better interpretation in such systems (Jones & Kuś, 2010). Despite the presence of the naphthalene ring systems, there are no significant *Cg*···*Cg* interactions. The shortest H···*Cg* contact is H7A···*Cg* (1 - *x*,1 - *y*,-*z*) 3.10 Å between layers, but this is both long and has a narrow angle (124°). We can conclude that the crystal packing of the title compound is dominated by the contacts involving bromomethylene groups.

### Experimental

The title compound was prepared as described by Rivera *et al.* (2001) by treatment of a solution of 2,3-bis[{[(1,1-dimethylethyl)dimethylsilyl]oxy}methyl]- 6,7-bis[(phenylmethoxy)methyl]-naphthalene in chloroform with gaseous HBr. The compound was obtained as a colourless microcrystalline solid. Yield: 87%. Crystals suitable for X-ray diffraction were grown by slow evaporation of a solution in EtOH/hexane/CH_2_Cl_2_. *M*.p. (decomp.) 230–231° C (lit. 230° C). ^1^H NMR (CDCl_3_): δ = 7.83 (s, 4H), 4.84 (s, 8H) p.p.m..

The title compound is poorly soluble (< 1 g/*L*) in most organic solvents at room temperature, but is much more soluble in aromatic solvents, such as toluene or chlorobenzene, upon reflux.

### Refinement

Hydrogen atoms were included at calculated positions using a riding model with aromatic C—H 0.95, methylene C—H 0.99 Å. The *U*(H) values were fixed at 1.2 × *U*_eq_(C) of the parent C atom.

### Crystal data

**Table:**

C_14_H_12_Br_4_	*Z* = 1
*M**_r_* = 499.88	*F*(000) = 236
Triclinic, *P*1	*D*_x_ = 2.254 Mg m^−^^3^
Hall symbol: -P 1	Melting point: 503 K
*a* = 6.6144 (2) Å	Mo *K*α radiation, λ = 0.71073 Å
*b* = 7.1770 (2) Å	Cell parameters from 9533 reflections
*c* = 8.7761 (3) Å	θ = 2.4–30.7°
α = 84.744 (3)°	µ = 10.91 mm^−^^1^
β = 78.251 (3)°	*T* = 100 K
γ = 64.555 (3)°	Prism, colourless
*V* = 368.32 (2) Å^3^	0.20 × 0.06 × 0.04 mm

### Data collection

**Table:**

Oxford Diffraction Xcalibur Eos diffractometer	2122 independent reflections
Radiation source: Enhance (Mo) X-ray Source	1716 reflections with *I* > 2σ(*I*)
graphite	*R*_int_ = 0.031
Detector resolution: 16.1419 pixels mm^-1^	θ_max_ = 30.0°, θ_min_ = 2.4°
ω–scan	*h* = −9→9
Absorption correction: multi-scan (*CrysAlis PRO*; Oxford Diffraction, 2009)	*k* = −10→10
*T*_min_ = 0.356, *T*_max_ = 1.000	*l* = −11→12
17701 measured reflections	

### Refinement

**Table:**

Refinement on *F*^2^	Primary atom site location: structure-invariant direct methods
Least-squares matrix: full	Secondary atom site location: difference Fourier map
*R*[*F*^2^ > 2σ(*F*^2^)] = 0.016	Hydrogen site location: inferred from neighbouring sites
*wR*(*F*^2^) = 0.034	H-atom parameters constrained
*S* = 0.92	*w* = 1/[σ^2^(*F*_o_^2^) + (0.0181*P*)^2^] where *P* = (*F*_o_^2^ + 2*F*_c_^2^)/3
2122 reflections	(Δ/σ)_max_ = 0.001
82 parameters	Δρ_max_ = 0.50 e Å^−^^3^
0 restraints	Δρ_min_ = −0.49 e Å^−^^3^

### Special details

**Table:**

Geometry. All e.s.d.'s (except the e.s.d. in the dihedral angle between two l.s. planes) are estimated using the full covariance matrix. The cell e.s.d.'s are taken into account individually in the estimation of e.s.d.'s in distances, angles and torsion angles; correlations between e.s.d.'s in cell parameters are only used when they are defined by crystal symmetry. An approximate (isotropic) treatment of cell e.s.d.'s is used for estimating e.s.d.'s involving l.s. planes.
Refinement. Refinement of *F*^2^ against ALL reflections. The weighted *R*-factor *wR* and goodness of fit *S* are based on *F*^2^, conventional *R*-factors *R* are based on *F*, with *F* set to zero for negative *F*^2^. The threshold expression of *F*^2^ > σ(*F*^2^) is used only for calculating *R*-factors(gt) *etc*. and is not relevant to the choice of reflections for refinement. *R*-factors based on *F*^2^ are statistically about twice as large as those based on *F*, and *R*- factors based on ALL data will be even larger.

### Fractional atomic coordinates and isotropic or equivalent isotropic displacement parameters (Å^2^)

**Table:**

	*x*	*y*	*z*	*U*_iso_*/*U*_eq_	
Br1	0.64988 (3)	−0.00387 (3)	0.24985 (2)	0.01628 (5)	
Br2	0.23208 (3)	0.72525 (3)	0.44061 (2)	0.01672 (5)	
C1	−0.0170 (3)	0.4100 (3)	0.02483 (19)	0.0108 (3)	
C2	0.0922 (3)	0.2877 (3)	0.14549 (19)	0.0122 (3)	
H2	0.0699	0.1672	0.1791	0.015*	
C3	0.2297 (3)	0.3393 (3)	0.21518 (19)	0.0111 (3)	
C4	0.2621 (3)	0.5223 (3)	0.16547 (19)	0.0110 (3)	
C5	0.1575 (3)	0.6419 (3)	0.04994 (19)	0.0112 (3)	
H5	0.1791	0.7633	0.0185	0.013*	
C6	0.3431 (3)	0.2029 (3)	0.34014 (19)	0.0136 (3)	
H6A	0.2495	0.1316	0.3945	0.016*	
H6B	0.3563	0.2876	0.4173	0.016*	
C7	0.4062 (3)	0.5854 (3)	0.24055 (18)	0.0135 (3)	
H7A	0.4559	0.6799	0.1704	0.016*	
H7B	0.5437	0.4620	0.2592	0.016*	

### Atomic displacement parameters (Å^2^)

**Table:**

	*U*^11^	*U*^22^	*U*^33^	*U*^12^	*U*^13^	*U*^23^
Br1	0.01360 (10)	0.01494 (10)	0.01724 (10)	−0.00349 (8)	−0.00312 (7)	0.00232 (7)
Br2	0.01615 (10)	0.01865 (11)	0.01468 (9)	−0.00544 (8)	−0.00321 (7)	−0.00581 (7)
C1	0.0099 (8)	0.0112 (9)	0.0093 (8)	−0.0031 (7)	0.0001 (6)	−0.0012 (6)
C2	0.0130 (8)	0.0120 (9)	0.0105 (8)	−0.0053 (7)	0.0000 (7)	−0.0003 (6)
C3	0.0085 (8)	0.0127 (9)	0.0087 (8)	−0.0016 (7)	−0.0006 (6)	−0.0008 (6)
C4	0.0092 (8)	0.0139 (9)	0.0097 (8)	−0.0050 (7)	0.0011 (6)	−0.0040 (6)
C5	0.0111 (8)	0.0104 (8)	0.0120 (8)	−0.0055 (7)	0.0012 (6)	−0.0020 (6)
C6	0.0111 (8)	0.0156 (9)	0.0115 (8)	−0.0033 (7)	−0.0017 (7)	0.0003 (7)
C7	0.0128 (8)	0.0177 (9)	0.0101 (8)	−0.0064 (7)	−0.0017 (7)	−0.0023 (7)

### Geometric parameters (Å, °)

**Table:**

Br1—C6	1.9801 (16)	C4—C7	1.493 (2)
Br2—C7	1.9806 (16)	C5—C1^i^	1.422 (2)
C1—C2	1.418 (2)	C2—H2	0.9500
C1—C1^i^	1.420 (3)	C5—H5	0.9500
C1—C5^i^	1.422 (2)	C6—H6A	0.9900
C2—C3	1.377 (2)	C6—H6B	0.9900
C3—C4	1.436 (2)	C7—H7A	0.9900
C3—C6	1.494 (2)	C7—H7B	0.9900
C4—C5	1.363 (2)		
			
C2—C1—C1^i^	118.85 (18)	C1—C2—H2	119.2
C2—C1—C5^i^	122.57 (15)	C4—C5—H5	119.0
C1^i^—C1—C5^i^	118.58 (19)	C1^i^—C5—H5	119.0
C3—C2—C1	121.68 (15)	C3—C6—H6A	109.6
C2—C3—C4	119.29 (15)	Br1—C6—H6A	109.6
C2—C3—C6	119.30 (15)	C3—C6—H6B	109.6
C4—C3—C6	121.41 (15)	Br1—C6—H6B	109.6
C5—C4—C3	119.70 (15)	H6A—C6—H6B	108.1
C5—C4—C7	119.52 (15)	C4—C7—H7A	109.6
C3—C4—C7	120.77 (15)	Br2—C7—H7A	109.6
C4—C5—C1^i^	121.90 (15)	C4—C7—H7B	109.6
C3—C6—Br1	110.35 (11)	Br2—C7—H7B	109.6
C4—C7—Br2	110.18 (11)	H7A—C7—H7B	108.1
C3—C2—H2	119.2		
			
C1^i^—C1—C2—C3	0.0 (3)	C6—C3—C4—C7	−1.7 (2)
C5^i^—C1—C2—C3	179.24 (16)	C3—C4—C5—C1^i^	−0.5 (2)
C1—C2—C3—C4	0.5 (2)	C7—C4—C5—C1^i^	−179.53 (15)
C1—C2—C3—C6	−179.09 (15)	C2—C3—C6—Br1	92.22 (16)
C2—C3—C4—C5	−0.2 (2)	C4—C3—C6—Br1	−87.36 (16)
C6—C3—C4—C5	179.34 (15)	C5—C4—C7—Br2	99.15 (16)
C2—C3—C4—C7	178.76 (15)	C3—C4—C7—Br2	−79.86 (17)

Symmetry codes: (i) −*x*, −*y*+1, −*z*.

### Hydrogen-bond geometry (Å, °)

**Table:**

*D*—H···*A*	*D*—H	H···*A*	*D*···*A*	*D*—H···*A*
C6—H6A···Br2^ii^	0.99	2.96	3.7967 (17)	143
C6—H6A···Br2^iii^	0.99	2.98	3.7359 (16)	134
C5—H5···Br1^iv^	0.95	3.11	3.9399 (16)	147

Symmetry codes: (ii) *x*, *y*−1, *z*; (iii) −*x*, −*y*+1, −*z*+1; (iv) −*x*+1, −*y*+1, −*z*.

### Table 2 Bromine–bromine and related contacts and angles (Å, °)

*Cg* is the centroid of the C1–C5,C1(-x, 1-y, -z) ring.

**Table:**

System C—Br···Br—C or C—Br···*Cg*	Br···Br or Br···*Cg*	C—Br···Br (or C—Br···*Cg*), Br···Br—C	Operator
C6—Br1···Br2—C7	3.8972 (3)	76.45 (5), 134.79 (5)	1-x, 1-y, 1-z
C7—Br2···Br2—C7	3.8873 (4)	134.93 (5) × 2	-x, 2-y, 1-z
C7—Br2···Br2—C7	3.8913 (4)	76.72 (5) × 2	1-x, 1-y, 1-z
C6—Br1···*Cg*	3.89	158	1+x, -1+y, z
